# Neurology and vaccinations: considerations in the context of COVID-19/SARS-COV-2

**DOI:** 10.1007/s00415-021-10513-w

**Published:** 2021-03-15

**Authors:** M. Cauchi, N. P. Robertson

**Affiliations:** Department of Neurology, Division of Psychological Medicine and Clinical Neuroscience, University Hospital of Wales, Heath Park, Cardiff, CF14 4XN UK

As the world embarks on the largest vaccination program in history, it is worth reflecting on the huge logistical effort this will involve. Vaccination remains the strongest tool in the public health armamentarium for prevention of infectious diseases, but achieving herd immunity will require population vaccination rates of around 70%. Apart from the huge clinical delivery and manufacturing efforts required to achieve this, successful inoculation efforts will also depend on addressing vaccine hesitancy and misinformation.

Some vaccines have previously been associated with the occurrence of rare, immune-mediated adverse events, although these can sometimes be difficult to separate from the overall infection burden at the time. However, we will need to remain vigilant for those events which may have occurred as a consequence of Covid-19 vaccination, as even very low-frequency events may have significant health burdens in the context of the number of anticipated vaccinations. It is important to note that study data available to date for the Covid-19 vaccination program remains reassuring.

In this month’s journal club, we review some articles that explore the association of neurological complications associated with large-scale vaccination events, as well as immune response to vaccination in the context of a modern therapeutic agent. The first is a phase IIIb trial assessing the impact of ocrelizumab on vaccine response in people with multiple sclerosis (pwMS). The second is a case–control study that set out to investigate the hypothesis that vaccination is a risk factor for MS, and the last is a study reporting on the association between influenza vaccination and Guillain-Barré syndrome (GBS).

## Effect of ocrelizumab on vaccine responses in patients with multiple sclerosis: the VELOCE study

It is thought that selected disease-modifying therapies (DMTs) for MS may lead to a significant increase in risk of infection because of their mode of action, and it is established that infections can be associated with high morbidity as well as disease progression. However, patients on DMTs may mount a lower immune response to vaccination, in particular those on B cell-depleting drugs. This study is a phase IIIb trial assessing the impact of Ocrelizumab-induced B cell depletion on vaccination response in pwMS. Ocrelizumab is a CD20-selective humanized monoclonal antibody licenced for use in relapsing and primary progressive MS, administered in 6 monthly infusions.

The study recruited 68 participants with MS receiving ocrelizumab 600 mg (as a first dose) and 34 in a control group (either on interferon beta or no DMT) vaccinated with tetanus toxoid (TT)-containing vaccine, Pneumovax [23-valent pneumococcal polysaccharide vaccine (23-PPV)], and keyhole limpet hemocyanin (KLH). The ocrelizumab group were randomized to either receiving Prevnar (a 13-valent conjugate pneumococcal vaccine) as a booster 4 weeks after Pneumovax (OCR1 group, *n* = 33), or an influenza vaccine (OCR2 group, *n* = 35). The control group also received the influenza vaccine. Vaccinations were administered 12 weeks after ocrelizumab infusions.

Primary endpoint was the proportion of patients with positive response to TT-containing booster (defined as an antibody titre ≥ 0.2 IU/mL or ≥ fourfold increase in titre if pre-vaccination levels were ≥ 0.1 IU/mL) 8 weeks after vaccination. In the ocrelizumab group, positive response rate to TT vaccine was 23.9% at 8 weeks (vs 54.5% in the control group). Response to ≥ 5 serotypes in the 23-PPV at 4 weeks was 71.6 vs 100% in the ocrelizumab and control groups, respectively. Response to the influenza vaccine was reduced in the OCR2 (55.6–80.0%) vs the control group (75.0–97.0%). Humoral response to KLH (a highly immunogenic T cell-dependent antigen used commonly in immunotoxicological studies) was also decreased in the ocrelizumab group. No safety issues were identified in either group. The study also noted that ocrelizumab-treated patients maintain their pre-treatment specific humoral immunity to common viral and bacterial antigens.

***Comment:*** This study demonstrated that non-live vaccines given at least 12 weeks after Ocrelizumab infusion can still result in an appropriate, although attenuated, immune response and provide sustained seroprotection in pwMS. The 12-week interval was chosen to investigate the effect of complete peripheral B cell depletion on the immune response to vaccinations, due to the long half-life of the anti-CD20 antibody.

The authors conclude that vaccination should be recommended for all patients on ocrelizumab, including with the annual seasonal influenza vaccine as an appropriate protective humoral response can be expected. Live or live-attenuated vaccines were not studied and are not recommended following ocrelizumab.

Bar-Or A et al. Neurology. 2020 Oct 6;95(14):e1999–e2008. https://doi.org/10.1212/WNL.0000000000010380.

## A large case–control study on vaccination as risk factor for multiple sclerosis

There remains considerable controversy on whether vaccination can trigger disease progression or exacerbation in MS or be a causative factor in disease initiation. Studies to date have shown conflicting results. The aim of this case–control study was to investigate the hypothesis that vaccination is a risk factor for MS using retrospective analysis of ambulatory claims data of 223,035 participants < 70 years old (12,262 patients with MS and 210,773 controls), held by the Bavarian Association of Statutory Health Insurance Physicians (BASHIP).

A cohort of newly diagnosed pwMS and two control cohorts of participants diagnosed with other autoimmune diseases (Crohn’s disease and psoriasis) were identified using registered ICD-10 diagnosis codes. Additional controls without these diagnoses (*n* = 83,610) were matched to the MS cohort in a 5:1 ratio. Inclusion in the MS cohort required a recorded neurologist visit and no diagnosis of a clinically isolated syndrome (CIS) before or after MS diagnosis. The authors focused on the 5-year period prior to diagnosis, during which any recorded reimbursement claim for vaccination, or combinations of vaccination, was included. Association between vaccination and MS was assessed using unconditional logistic regression models and odds ratios. The analysis was repeated for various time frames (quartiles) in the 5-year period prior to diagnosis.

There was a lower odds ratio for MS in participants with any vaccination, consistent across all studied time frames and control cohorts. A low rate of vaccination in the months preceding an MS diagnosis was also identified, arguing against a major role of vaccination in triggering relapses. The negative association of vaccines with MS was present for all reported vaccinations and specifically for influenza, hepatitis B, and Tick-Borne Encephalitis.

***Comment:*** This large case–control study demonstrates a lower rate of vaccination in the 5 years preceding MS onset, supporting the hypothesis that vaccination is not a factor in MS onset. The size of the cohort is a particular strength. Although the study was anchored to time of diagnosis, the authors used strict definitions of MS (requiring less than one neurologic consultation or MRI in the 5-year period, and no diagnosis of optic neuritis). The results of this study provide further evidence against the role of vaccination as an aetiological factor for MS.

Hapfelmeier A et al. Neurology. Aug 2019;93(9):e908–e916. https://doi.org/10.1212/WNL.0000000000008012.

## Guillain–Barré syndrome after high-dose influenza vaccine administration in the United States, 2018–2019 season

Following the 1976 US influenza vaccination program, when an increased risk of GBS was identified, seasonal influenza vaccine programs have been actively surveyed for adverse events. Most of these studies have found either no risk or a small increase in risk of around 1–3 additional cases per million vaccinees; a risk significantly lower than the risk of GBS caused by influenza itself.

This study investigated the risk of GBS during the 2018–2019 season using the Medicare database after the Vaccine Safety Datalink (VSD) project identified a possible early statistical signal. The VSD then conducted weekly rapid cycle analysis to monitor risk of GBS following seasonal high-dose influenza vaccination (IIV3-HD). End-of-season nonsequential chart-confirmed self-controlled risk interval analyses (SCRI) involved 646,996 IIV3-HD vaccinations and yielded a RR of 1.00 (95% CI 0.06–15.99). The observed number of GBS cases occurring in the 1–42 days after IIV3-HD administration was also compared with post-vaccination rate in prior influenza seasons (2012–2016), with no increased risk identified.

An early and end-of-season SCRI was then conducted among 14,437,945 (all seasonal vaccines combined of which 8,667,640 were IIV3-HD) influenza-vaccinated Medicare beneficiaries aged ≥ 65 years to compare number of GBS cases in days 1–42 post-vaccination (risk window) with that in days 43–84 post-vaccination (control window). In the end-of-season analyses, there was no statistically significant increase in GBS risk observed in either the 8–21 days post-vaccination (OR 1.64; 95% CI 0.92–2.91) or 1–42 days post-vaccination (OR 1.12; 95% CI 0.70–1.79) risk windows. These estimates did not change after seasonality adjustments.

In summary, no statistically significant increased risk of GBS following IIV3-HD was identified for the 2018–2019 season.

***Comment:*** Although this study did not exclude an association between 2018 and 2019 IIV3-HD and GBS, it was able to determine that if such a risk existed, it was reassuringly low. Studies have repeatedly demonstrated a stronger association between a preceding viral illness and GBS; and the benefits of influenza vaccination appear to greatly outweigh the risk.

Perez-Vilar S et al. J Infect Dis. 2021 Feb 13;223(3):416–425. https://doi.org/10.1093/infdis/jiaa543.

